# Potential of Persimmon Dietary Fiber Obtained from Byproducts as Antioxidant, Prebiotic and Modulating Agent of the Intestinal Epithelial Barrier Function

**DOI:** 10.3390/antiox10111668

**Published:** 2021-10-22

**Authors:** Julio Salazar-Bermeo, Bryan Moreno-Chamba, María Concepción Martínez-Madrid, Domingo Saura, Manuel Valero, Nuria Martí

**Affiliations:** 1 Instituto de Investigación, Desarrollo e Innovación en Biotecnología Sanitaria de Elche (IDiBE), Universidad Miguel Hernández de Elche, 03202 Alicante, Spain; julio.salazar@goumh.umh.es (J.S.-B.); bryan.morenoc@umh.es (B.M.-C.); dsaura@umh.es (D.S.); nmarti@umh.es (N.M.); 2 Departamento de Agroquímica y Medio Ambiente, Universidad Miguel Hernández de Elche, 03312 Alicante, Spain; c.martinez@umh.es

**Keywords:** *Diospyros kaki*, antioxidant activity, in vitro digestion, probiotic bacterial fermentation, bioactive compounds

## Abstract

Appropriate nutrition targets decrease the risk of incidence of preventable diseases in addition to providing physiological benefits. Dietary fiber, despite being available and necessary in balanced nutrition, are consumed at below daily requirements. Food byproducts high in dietary fiber and free and bonded bioactive compounds are often discarded. Herein, persimmon byproducts are presented as an interesting source of fiber and bioactive compounds. The solvent extraction effects of dietary fiber from persimmon byproducts on its techno- and physio-functional properties, and on the Caco-2 cell model after being subjected to in vitro gastrointestinal digestion and probiotic bacterial fermentation, were evaluated. The total, soluble, and insoluble dietary fiber, total phenolic, carotenoid, flavonoid contents, and antioxidant activity were determined. After in vitro digestion, low quantities of bonded phenolic compounds were detected in all fiber fractions. Moreover, total phenolic and carotenoid contents, as well as antioxidant activity, decreased depending on the extraction solvent, whereas short chain fatty acids production increased. Covalently bonded compounds in persimmon fiber mainly consisted of hydroxycinnamic acids and flavanols. After probiotic bacterial fermentation, few phenolic compounds were determined in all fiber fractions. Results suggest that persimmon’s dietary fiber functional properties are dependent on the extraction process used, which may promote a strong probiotic response and modulate the epithelial barrier function.

## 1. Introduction

Appropriate nutrition targets decrease the risk of incidence of preventable diseases in addition to providing physiological benefits [1,2,3]. The development of food products containing physiologically bioactive molecules capable of maintaining and/or improving beneficial long-term effects may contribute to achieve these objectives. For instance, persimmon fruit (*Diospyros kaki* Thunb.), a widespread cultivar in the south of Spain and China, has been found to provide a significant amount of bioactive compounds with physiological benefits [4,5].

The main compounds in persimmon have been reported to be polyphenols (gallic acid, coumaric acid, epicatechin, kaempferol, and ellagic acid), carotenoids (neoxanthin, antheraxanthin, lutein, zeaxanthin, β-carotene, and lycopene), and polysaccharides (pectin, cellulose, hemicellulose) [6,7,8]. Studies have shown the hypocholesterolemic, hypolipidemic, anti-atherogenic, anti-obesity, antidiabetic, antioxidant and antiviral effects of persimmon fruits and leaves in in vitro and animal models [9,10,11]. Due to its fast ripening, persimmon fruits are rapidly processed and generate a high amount of byproducts. The abundance of dietary fiber (DF) in fruit byproducts makes them attractive for second generation bio-refining and promoting the valorization of agricultural byproducts that are not part of value chain of the industry [12]. Obtained from byproducts, DF may help target disease prevention and the reduction of risks, such as atherosclerosis, cardiovascular disease, and colorectal cancer [13].

Fiber intake in Western populations reaches fifty percent of the daily recommended value of DF [13]. The beneficial effects of DF are directly influenced by their mechanical properties, known as physio-functional and techno-functional properties. Due to gastrointestinal degradation resistance, DF is determinant for gut microbiota ecology, diversity, and function. Metabolites produced from beneficial gut microbiota (e.g., Firmicutes and Bacteroidetes) consist of short chain fatty acids (SCFAs), such as acetate, propionate, and butyrate. SCFAs are metabolized by epithelial cells and increase the production of anti-inflammatory cytokines, influence cellular metabolism in colonocytes, fibroblasts, and adipocytes [14,15,16]. Diets low in fermentable substrates result in a thinner mucus layer lining the gut lumen, increasing the susceptibility to the infection of intestinal epithelial cells [17]. To our knowledge, this is the first study that evaluates the extraction effects of persimmon DF after gastrointestinal digestion and fermentation in human cell lines.

The enrichment of fiber content in food matrices throughout untreated fruit byproducts could have adverse effects on the glycemic index of some enriched foods due to the sugar content. Moreover, it could alter expected food sensory profiles. The treatment of byproducts with appropriate solvents may provide a DF with valuable bioactive compounds, while extracting other compounds of interest, such as carotenoids and phenolics [7]. Studies have reported that byproducts still retained a substantial amount of covalently bonded bioactive compounds, and their antioxidant activities, such as radical scavenging activity [18]. These remnants, after gastrointestinal digestion and when available, may be a key point for beneficial and pathogenic bacteria, health, and well-being. However, byproducts generated by food manufacturers may not be appropriate for immediate upcycling without previous treatments. The aim of this research was to evaluate the solvent extraction effects of DF from persimmon byproducts on its functional properties and safety on human epithelial cells after being subjected to in vitro gastrointestinal digestion and beneficial gut bacterial fermentation.

## 2. Materials and Methods

### 2.1. Chemicals and Reagents

Ethanol (99.5%), methanol (99.9%), acetone (99.9%), sulfuric acid (96%), petroleum ether (40–60 °C), acetic acid glacial (99.8%), acetonitrile (99.9%), hydrochloric acid (37%) and sodium hydroxide (40%) were obtained from PanReac (Barcelona, Spain). α-Amylase, pepsin, pancreatin, porcine bile extract, electrolytes (CaCl_2_, KCl, KH_2_PO_4_, NaHCO_3_, MgCl_2_ and (NH_4_)_2_CO_3_), Folin Ciocalteu’s reagent, crystal violet staining, 3-(4,5-dimethylthiazol-2-yl)-2,5-diphenyltetrazolium bromide, dimethyl sulfoxide, phosphate buffered saline solution and reference reagents for identification of phenolics and SCFAs were purchased from Sigma-Aldrich (Madrid, Spain). Microbial culture media was obtained from Scharlab (Barcelona, Spain), while pure culture probiotic strains were purchased from Spanish Type Culture Collection (CECT) (Valencia, Spain). Cell culture medium and reagents were obtained from Fisher Scientific (Madrid, Spain).

### 2.2. Plant Material

*Diospyros kaki* Thumb. from the ‘Rojo Brillante’ variety were selected based on uniformity from a local market (Alicante, Spain). The fruits were in the orange ripening stage at 15 ± 2° Brix, grouped into batches, washed, disinfected, the stem was separated, and the fruits were cut and processed at pilot scale; the juice was filtered, and byproducts made up by pulp and peels were collected and stored at −18 °C.

### 2.3. Solvent Assisted Extraction (SAE)

The byproduct was mixed with 70% (*v*/*v*) solvent:water solution at a 5:1 (*v*/*w*) ratio, then the mixture was heated at 60 °C and stirred for 15 min at 3600 rpm. Based on the solvent applied for the assisted extraction, the fractions obtained were: Persimmon Fiber Aqueous Extraction (PFAE), Persimmon Fiber Acetonic Extraction (PFAC), and Persimmon Fiber Ethanolic Extraction (PFEE). Final mixtures were filtered and freeze-dried before use.

### 2.4. Physicochemical Analysis

Total DF (TDF), insoluble DF (IDF), soluble DF (SDF), moisture, ash, and crude protein content were determined according to the Association of Official Analytical Chemist official enzymatic-gravimetric method 991.43 [19]. All analyses were carried out in triplicate.

### 2.5. Techno-Functional Properties

The water absorption activity (WAA) of the DF fractions was measured as described by [20] and expressed as the volume of water held by DF fractions after centrifugation. The water-holding activity (WHA) was expressed as the weight of water held by the weight corresponding to the DF fractions [21]. The swelling activity (SA) of the DF fractions was assessed according to [22] and expressed as milliliters of DF per gram of the DF samples. The oil-holding activity (OHA) of the DF fractions was evaluated and expressed as the weight of oil held by the weight of the DF samples (g/g) [21]. The emulsifying activity (EA) and emulsion stability (ES) were expressed as the volume of emulsion formed by the DF samples and the percentage (%) of emulsified and stable fraction, respectively [23]. The gel formation activity (GFA) was determined according to [24]. The DF solutions were expressed as the minimum percentage (*w/v*) of DF with GFA.

### 2.6. Physio-Functional Properties

The bile-holding activity (BHA) of the DF fractions was measured as the weight of porcine bile held by the DF fractions [25], while the fat/oil binding (FOB) capacity was measured as the adsorption capacity of fats on the DF matrix after simulated conditions of digestion. The FOB capacity of each fraction was expressed as grams of oil held by grams of DF (g/g).

### 2.7. In Vitro Gastrointestinal Digestion

The in vitro gastrointestinal digestion of the three treatments was simulated following the INFOGEST methodology described by [26] adapted for DF matrices. Simulated digestion fluids were prepared and sterilized prior digestion. A sample of 0.5 g of each extracted fraction and a control were subjected to three phases: oral, gastric, and intestinal at 37 °C. The pH, time, and simulated digestion fluids were adjusted for each phase. Afterwards, digested fractions were stored at −80 °C until further use.

### 2.8. Probiotic Fermentation Process (PFP)

To test the biological potential of extracted fiber and the effects of the digestion process, fermentation was performed before and after the in vitro gastrointestinal digestion on selected beneficial host microorganisms according to the methodologies established by [27,28,29,30]. A 10 mL volume of each homogenized fraction and control were centrifuged at 948× *g*, 10 min at 4 °C, and the supernatants and pellets were separated. Then, 100 mg of the pellet and 50 µL of the supernatant were combined and mixed with a 150 µL inoculum of four human host beneficial bacteria strains.

The strains were selected based on their microbiome diversity, health implications, and to test the production of SCFAs without the interference of other metabolites. Bacterial suspensions of *Bifidobacterium bifidum* CECT 870, *Lactobacillus casei* CECT 475, *Lactococcus lactis* subsp. *lactis* CECT 185, and *Streptococcus salivarius* subsp. *thermophilus* CECT 7207 in 5 mL sterilized distilled water at a concentration of 10^7^ CFU/mL were used. Homogenized mixtures were incubated at 37 °C for 48 h in aerobiosis (*L. casei* and *S. salivarius*) and anaerobiosis (*B. bifidum* and *Lc. lactis*). Afterwards, fermented samples were centrifuged (pellet and soluble fraction) and stored at −80 °C until further analysis.

### 2.9. Cell Culture

Human epithelial colorectal adenocarcinoma cell line (Caco-2; American Type Culture Collection, HTB-37) was used in this study as a human intestinal barrier model. The cell line was grown and maintained in Dulbecco’s Modified Eagle Medium (DMEM) supplemented with 10% heat-inactivated fetal bovine serum (SBF), 1% penicillin/streptomycin, 1% of nonessential amino acids, and N-2-hydroxyethylpiperazine-N-2-ethane sulfonic acid (HEPES) 1 M solution. Cells were maintained (37 °C and 5% CO_2_ atmosphere) between 15–20 passages before assays. At every passage (70–80% confluence), the cells were rinsed with phosphate buffered saline, pH 7.2, supplemented with 1 mM 2,2′,2″,2‴-(Ethane-1,2-diyldinitrilo)tetra-acetic acid (PBS-EDTA solution), trypsinized with 0.25% trypsin, and trypsin-neutralized with new completed DMEM before being diluted.

#### 2.9.1. Cell Viability

After the fermentation of the digested fractions, the obtained product was tested on the Caco-2 cells monolayer. Aliquots of 200 µL of DMEM with 1.5 × 10^4^ Caco-2 cells (15–20 passages) were seeded in 96-well plates. After monolayer formation, plates were incubated (37 °C, 5% CO_2_) for one week. The culture media was changed every other day with a new complete medium. An 8-day model was used for the viability assessment. Before the assay, the media was discarded from plates and media containing post digestion and fermentation metabolites was added. 200 µL of pure fermented fractions were added into the first row; then, they were two-fold diluted in the completed medium. The plates were incubated (37 °C, 5% CO_2_) for 24 h. Two methods for cell viability were performed.

The crystal violet staining (CVS) assay was used to determine viable adhered cells [31]. The medium was discarded and 100 µL of DMEM with 0.5% CV was added to every well for 20 min at 37 °C. The CV solution was discarded from the plates, rinsed with pure water, and dried at room temperature for 2 h. 100 µL of pure methanol was added to the wells. Optical density (OD) at 590 nm was recorded by the microplate reader Cytation™ 3 Cell Imaging Multi-Mode (BioTek, Winooski, VT, USA).

The (3-(4,5-Dimethylthiazol-2-yl)-2,5-diphenyltetrazolium bromide (MTT) assay was also performed [32]. Culture media from 96-well plates was discarded. Then, 100 µL of DMEM with 5 mg/mL of MTT solution was added. The plates were incubated (37 °C, 5% CO_2_) for 3 h. Then, medium was discarded, and plates were dried at room temperature for 2 h. A volume of 100 µL of pure dimethyl sulfoxide (DMSO) was added. The formazan production of viable cells was recorded at 550 nm using the Cytation 3 microplate reader. The percentage of viability of Caco-2 cells was determined comparing the viability of treated with untreated cells.

#### 2.9.2. Trans Epithelial Electrical Resistance (TEER) Response of Caco-2 Cell Monolayer

The effect of fermented products of DF on intestinal epithelial barrier function was also tested using Caco-2 cells [33,34]. 1 × 10^5^ cells (15–20 passages) were seeded into inserts of 0.4 µm pore size in 6-well plate. The culture media (DMEM) was changed every other day throughout 21 days of incubation. A 21-day model was used to study the TEER response during 8 h incubation with the fermented samples. The 21-day Caco-2 monolayers were rinsed twice with Hank’s Balanced Salt Solution (HBSS) with 1 M HEPES (pH 7.4).

TEER was measured using a Millicell ERS-2 for 8 h of incubation (37 °C and 54 rpm of shaking). Caco-2 cells were incubated with media from fermentation samples diluted in HBSS at a 1:1 ratio (2 mL in apical chamber and 3 mL in basolateral chamber of different plates). TEER was also measured in a blank (insert with HBSS with no cellular monolayer) and a control (monolayer with no fermented samples added). Monolayers with TEER values above 350 Ω cm^2^ were used for the assay. Fermented samples were diluted in HBSS to address their effect in Caco-2 monolayers without the influence of other nutrients. The assay was carried out by triplicate.

### 2.10. Determination of Biocompounds

#### 2.10.1. Sample Preparation

To hydrolyze bonded bio-compounds from DF fractions, 0.25–0.50 g from each undigested, digested and fermented DF fraction were separately mixed with 5 mL of NaOH (2 M) for 18 h at room temperature, samples were then acidified with HCl (resulting pH <2) and extracted with methanol (80%, *v*/*v*) three times. The extracted fractions were filtered through a 0.45 µm filter; vacuum dried and stored at −80 °C until further use. To assess extracted and metabolized compounds after the digestion and fermentation process, samples from the supernatant formed after in vitro digestion and bacterial fermentation were not hydrolyzed, as control.

#### 2.10.2. The Folin-Ciocalteu Reagent Assay

To determine the total phenolic content (TPC) of each fraction the Folin–Ciocalteu’s reagent was used [35,36], acknowledging its limitations as a reducing capacity assay [37,38]. 0.125 mL of the sample was mixed with 0.5 mL of distilled water and 0.125 mL of Folin-Ciocalteu reagent. After 6 min in darkness, 1.25 mL of (Na_2_CO_3_) was added and 1 mL of distilled water. After 1 h, the absorbance values were measured on the Cytation 3 microplate reader at 760 nm and compared to a standard curve of Gallic acid.

#### 2.10.3. Total Carotenoid Content (TCC)

The TCC was measured according to the method described by [39], with modifications. Approximately 5 g or 5 mL of each fraction was homogenized with 5 mL of petroleum ether, 2.5 mL of acetone and 2.5 mL of ethanol. The suspension was stirred for 30 min at 4 °C and centrifuged at 6000× *g* for 10 min at 4 °C. The supernatants were pooled, and 10 mL of water was added. Absorbance values were measured on the Cytation 3 microplate reader at 450 nm and compared to a standard curve of β-carotene.

#### 2.10.4. Total Flavonoid Content (TFC)

The TFC was measured according to [40]. In total, 1000 µL of a diluted sample (1:20 *v*/*v*) was mixed with 1000 µL of aluminum chloride (2%, *w/v* in methanol) the mixture was allowed to react for 10 min. Absorbance values were measured on the Cytation 3 microplate reader at 368 nm and compared to a standard curve of quercetin.

### 2.11. Recovery and Bioaccessibility Index of Free and Bonded Compounds

The recovery index and bio-accessibility index were identified for the analysis of the digestion process effect in the bio-compounds content [41]. The recovery index shows the quantity of phenolic compounds and carotenoids available in the fiber matrix after the in vitro digestion, comparing it with the total bio-compounds (free and bonded) present in each undigested fraction measured in the fiber matrix. The bio-accessibility index compares the total amount of bioactive compounds found after the digestion process in the intestinal phase with the amount in the supernatant from the digestion and fermentation process.

### 2.12. Antioxidant Activity

#### 2.12.1. The 2,2′-Azino-Bis(3-Ethylbenzothiazoline-6-Sulfonic Acid) (ABTS^•+^) Radical Cation-Based Decolorization Assay

The ABTS^•+^ radical scavenging activity was determined as described by [42] with some modifications. The ABTS^•+^ solution (4 mM) was prepared with potassium persulfate (2.45 mM) and diluted to an absorbance of 720 ± 20 at 734 nm 24 h beforehand. The reactions were performed by adding 200 µL of ABTS^•+^ solution to 20 µL of each extract solution. Absorbance values were measured on the Cytation 3 microplate reader at 734 nm after 6 min of incubation at room temperature and compared to a standard curve of Trolox (6-hydroxy-2,5,7,8-tetramethylchroman-2-carboxylic acid).

#### 2.12.2. The 2,2-Diphenyl-1-Picrylhydrazyl (DPPH) Radical-Based Assay

The DPPH free radical scavenging activity was determined as described by [43], with some modifications. A DPPH solution (0.06 mM) in methanol was prepared. The reactions were performed by adding 180 µL of DPPH solution to 20 µL of each extract solution. Absorbance values were measured on the Cytation 3 microplate reader at 515 nm after 20 min of incubation at room temperature and compared to a standard curve of Trolox.

### 2.13. High Performance Liquid Chromatography Analysis (HPLC-DAD)

Polyphenolic quantification of the most abundant compounds found in undigested, digested and fermented DF fractions was determined by HPLC-DAD. Briefly, A HPLC Agilent (Santa Clara, CA, USA) series 1200 instrument, equipped with a HPLC column Poroshell 120 SB-C18, 2.7 µm, 4.6 × 150 mm was used.

Phenolic compounds were analyzed with a flow rate elution of 0.7 mL/min. The mobile phases used were acetic acid in Milli-Q^®^ water (0.5:99.5, *v*/*v*) as solvent A, and acetonitrile as solvent B. The chromatograms were recorded at full range UV/vis spectrum. Quantification was executed by comparing UV absorption spectra and retention times of each compound based on linear curves of authentic standards injected in the same conditions.

SCFAs production from the fermentation process was determined by HPLC-DAD following the methodology described by [44]. A HPLC Agilent series 1100 instrument, equipped with a HPLC column Supelcogel C610H 30 cm × 7.8 mm was used. Organic acids were analyzed, in standard and sample solutions, with a flow rate elution of 0.5 mL/min. The mobile phase used was phosphoric acid in Milli-Q^®^ water (0.1:99.9 *v*/*v*). The chromatograms were recorded at 210 nm. Quantification of organic acids was executed by comparing UV absorption spectra and retention times of each compound based on linear curves of authentic standards injected in the same conditions.

### 2.14. HPLC Coupled to Electro-Spray Ion Trap Mass Spectrometry (HPLC-DAD-ESI-IT-MS^n^)

The solvent assisted extracted fractions were analytically characterized by HPLC-DAD-ESI-IT-MS^n^. A 1100 HPLC system with a G1315B diode array detector (Agilent, Waldbronn, Germany) coupled on-line to an Esquire 3000+ ion trap mass spectrometer (Bruker Daltonik, Bremen, Germany) with an atmospheric electro spray ionization (ESI-API) source.

MS-parameters were adjusted, mass spectra were recorded in negative polarity mode at a scan range of *m*/*z* 50–1100. at a scan rate of 13,000 Th/s (peak width = 0.6 Th, fwhm). Nitrogen was used as both drying and nebulizing gas at a flow rate of 9 L/min and a pressure of 45 psi, respectively. Nebulizer temperature was set at 365 °C, and a potential of −4500 V was applied on the capillary. Collision gas for induced dissociation was helium at a pressure of 4.9 × 10^−6^ mbar; mass spectra were obtained with an isolation width of 4.0 *m*/*z* for precursor ions and a fragmentation amplitude of 1.0 V.

Control of the system and data evaluation was achieved with ChemStation for LC version A.00.03 (Agilent) and Esquire software version 5.1 (Bruker), respectively. Column and HPLC settings were as detailed below. Identification of phenolics was accomplished by comparison of UV-vis absorption spectra, retention times, and mass spectra with those of authentic standards.

When standards were unavailable, pigments were tentatively identified by comparing their UV-vis absorption spectra and mass spectral behavior with in-lab spectral library, data published previously and databases available [8,45].

### 2.15. Statistical Analysis

All experiments were carried out in triplicate and the results were expressed as mean values ± standard error (SE). Data obtained for each test was analyzed by means of a one-way ANOVA test. Tukey’s and Dunnett’s post hoc tests were applied for comparisons of means; differences were considered significant at *p* < 0.05. Statistical analyses were carried out using the statistical package GraphPad Prism 8.0.2. Correlation analysis was performed between physicochemical, techno-functional, and physio-functional properties using Pearson correlation analysis.

## 3. Results

### 3.1. Physicochemical Analysis

Physicochemical parameters studied in all treatments (Table 1) showed discreet differences influenced by the solvent applied (*p* < 0.05). In the case of protein content, all fractions analyzed presented low quantities with values ranging between 0.0002 to 0.0021 g/g of sample, which was probably related to the SAE treatment and the low nitrogen content in persimmon fruits.

**Figure 1:**
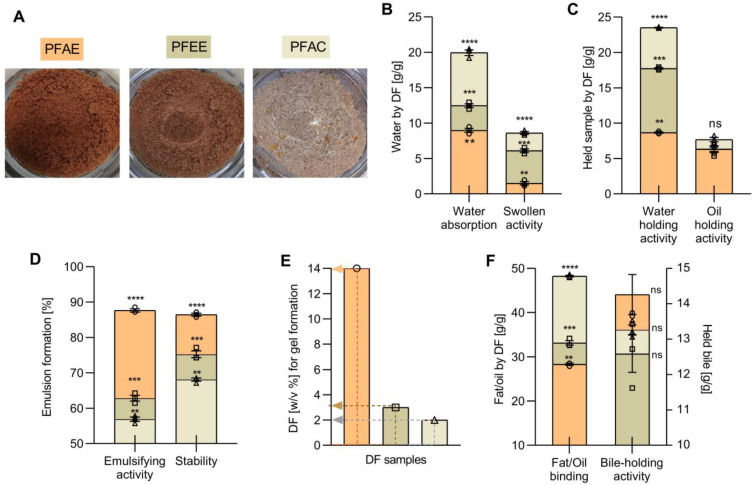
(**A**) Dietary fiber (DF) obtained from persimmon byproduct by aqueous extraction (PFAE, ○), ethanolic extraction (PFEE, □) and acetonic extraction (PFAC, **△**). Solvent assisted extraction (SAE) effect on techno-functional properties of persimmon fiber (PF); (**B**) hydration properties, (**C**) holding properties; (**D**) emulsifying properties, and (**E**) gel formation activity (GFA). (**F**) Solvent effect on physio-functional properties of PF (**** *p* < 0.0001, *** *p* < 0.001, ** *p* < 0.01, ns *p* > 0.05, ANOVA with Tukey’s post hoc test).

PFAC fractions (Figure 1A) showed the highest TDF content (0.94 ± 0.08), while no significant differences were observed between PFAE and PFEE (*p* < 0.05). The IDF content of all treatments was not affected by the solvent applied during extraction, as a result, no significant differences in IDF content were observed (*p* < 0.05). Treatments exhibited a higher IDF than SDF. On the other hand, SDF content was significantly affected (*p* < 0.05) where PFAC showed the highest yield in soluble polysaccharides (0.30 ± 0.16). SDF/IDF ratios for PFAE, PFEE, PFAC were 1:4, 1:6, and 1:3, respectively.

### 3.2. Techno-Functional Properties

Figure 1B,C show the results obtained for hydration (WAA and SA) and holding properties (WHA and OHA) from each treatment. The PFAC fraction showed the highest WAA, SA, WHA and OHA. Absorption, holding, and swelling capacities varied significantly, were influenced by the treatment (*p* < 0.05), and strongly related to the TDF, SDF and IDF content. WHA showed a positive correlation with TDF (r = 0.93) and SDF (r = 0.94), as well as WAA with SDF (r = 0.77) and TDF (r = 0.99), similarly to SA with SDF (r = 0.95) and TDF (r = 0.92). As for OHA, no significant differences were observed between treatments (*p* < 0.05). However, PFAC showed the highest value with a modest (r = 0.73) correlation between OHA and IDF.

Regarding emulsifying properties (Figure 1D), significant differences were obtained (*p* < 0.05), where PFAE showed the highest values for both emulsifying properties. The EA and ES were correlated with both SDF (r = 0.99), (r = 0.93) respectively, and TDF (r = 0.83), (r = 0.94) respectively; the decrease of these values influenced a lower percentage in PFAC and PFEE. Regarding the GFA (Figure 1E), the lowest amount of DF necessary to form a gel was recorded in PFAC which also reported the highest difference (*p* < 0.05); GFA was strongly related with the presence of SDF (r = 0.99).

### 3.3. Physio-Functional Properties

BHA (Figure 1F) was discreetly influenced by the treatments (*p* < 0.05), the highest values for PFAE and an inverse correlation with SDF (r = − 0.90) were denoted. In addition, the FOB of extracted fiber fractions, which is an essential parameter in the characterization of functional DF, showcased significant differences observed among treatments (*p* < 0.05); PFAC showed the highest FOB value (*p* < 0.05) and a strong relation with the TDF content (r = 0.99).

### 3.4. Recovery and Bioaccessibility of Free and Bonded Biocompounds

**Figure 2:**
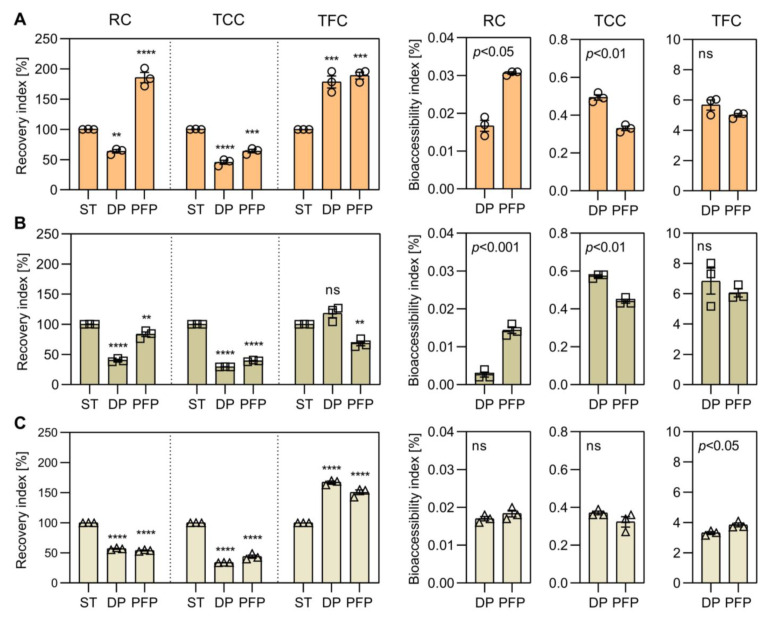
Recovery and bio-accessibility indexes of bonded total phenolic (TPC), total carotenoid (TCC) and total flavonoid content (TFC) in persimmon fiber (PF) after solvent treatment (ST), in vitro digestion (DP) and probiotic fermentation (PFP) processes. Recovery and bio-accessibility of bonded bio-compounds in PF obtained by (**A**) aqueous extraction (PFAE, ○), (**B**) ethanolic extraction (PFEE, □), and (**C**) acetonic extraction (PFAC, **△**) (recovery: **** *p* < 0.0001, *** *p* < 0.001, ** *p* < 0.01, ns *p* > 0.05, ANOVA with Dunnett’s post hoc test; bio-accessibility: *p* < 0.5, ANOVA with *t*-test post hoc).

Regarding to the recovery of phenolics, all fractions showed statistical differences after the digestion process; whereas PFAE (Figure 2A) and PFAC (Figure 2C) showed a similar reduction in their indexes, PFEE (Figure 2B) had a significant decrease on the TPC (*p* < 0.05). After the PFP, all fractions showed differences in the TPC recovery index. PFAE remained higher than the other fractions while PFEE and PFAC had a significant reduction after fermentation (*p* < 0.05) of the digested fraction. The bio-accessibility indexes of bonded phenolic compounds were relatively low (<10%) for all fractions.

PFAE showed the highest bio-accessibility only after the fermentation process (*p* < 0.05). Of the phenolic group, flavonoid recovery was high in PFAE and PFAC after digestion but in lower concentrations than other phenolic compounds. Their indexes decreased significantly after fermentation in PFAC and PFEE fractions (*p* < 0.05).

The recovery and bio-accessibility of TCC and TFC showed a similar behavior than that observed for TPC after digestion (Figure 2). Fractions decreased more than 50% their values and PFAE fraction showed the highest index (*p* < 0.05). Both PFAE and PFAC fractions increased their recovery index over the digested fraction in the fermented fraction; both PFEE and PFAC showed a similar behavior and indexes (*p* < 0.05) after digestion and fermentation.

### 3.5. Antioxidant Activity

As regards to the antioxidant activity (Table 2) provided by the bio-compounds presented in the fiber matrix, PFAE fraction from undigested fiber showed the highest activity (*p* < 0.05) followed by the PFAC and PFEE highlighting the dependence on the solvent applied for the extraction process. However, the pellet formed after digestion showed a lower antioxidant activity than the soluble fraction, which displays a complex interaction between the extracted supernatant and the fiber matrix that formed the pellet.

### 3.6. Phenolic Profile

In total, 23 phenolic compounds were identified (Table 3). Among the most abundant hydroxycinnamic acids, hydroxybenzoic acids, flavonols, flavanols, flavones, tannins, and stilbenes. Fiber bounded phenolic compounds found in persimmon belonged to the group of hydroxybenzoic acid derivatives. Retention time, MS, and UV-vis characteristics of compounds peaks were similar to those of the authentic standards and agreed with our previous results and reported elsewhere in persimmon [8,45,46]. Few differences in the phenolic profile were observed from treatments, while a greater profile of fiber bonded compounds was observed.

From the hydroxybenzoic acids subgroup, compound No. **1** displayed a fragmentation pattern at *m*/*z* 126 and eluted at 7.7 min, presented absorbance maxima at 280 nm in accordance with literature, and was reported as Gallic acid. This compound was reported in all PFAE, PFAC, and PFEE fractions before and after digestion and fermentation; however, it was not observed in high intensity in the supernatant after the digestion process. Compounds **3** and **5** were ellagic acid and salicylic acid at *m*/*z* 303 and 301, which corresponded with authentic standards and were observed in all fractions before the fermentation process.

From the hydroxamic acid group, compounds **2**, **4**, **6**, **22** and **23** corresponded to *p*-coumaric acid at *m*/*z* 164, sinapic acid at *m*/*z* 209, dicaffeoylquinic acid at *m*/*z* 514, ferulic acid glucoside at *m*/*z* 355, and *p*-coumaroyl tartaric acid at *m*/*z* 297. The product ions observed in the ESI(-)-MS2 experiment were in agreement with previous findings [8,47,48]. *p*-Coumaric acid was observed as bonded in the fractions before and after digestion and extractable in the PFAC fraction after the digestion process. Likewise, synaptic acid was also observed in the extractable fraction in the PFAE and PFEE, whereas dicaffeoylquinic acid and ferulic acid glucoside were only observed to be bonded and was detected in low intensity after the digestion process. Similarly, *p*-coumaroyl tartaric acid was found bonded to the fiber matrix and only detectable in hydrolyzed, digested, and fermented samples.

Flavonols detected at peaks 7, 13, 14, 18, and 19 with retention times of 20.9, 24.2, 25.2, 27.2, and 27.6 min were identified according to their main ions and fragmentation patterns. Spinacetin 3-*O*-(2″-*p*-coumaroylglucosyl) (1->6)-[apiosyl(1->2)]-glucoside at *m*/*z* 1021, was found in all fractions (PFAE, PFAC, PFEE) during the digestion and fermentation process; kaempferol-7-glucoside at *m*/*z* 446, was detected bonded and released to the supernatant after digestion (PFAE, PFAC, PFEE) and fermentation process (PFAE). Quercetin glucoside isomers were identified at *m*/*z* 783 and found bonded and released to the supernatant after the digestion fermentation process in all fractions. Kaempferol 3-*O*-glucosyl-rhamnosyl-galactoside at *m*/*z* 754 was detected free and bonded in all extracted fractions.

Isoflavonoids, compounds **8** and **20** eluted at RT 21.6- and 28.5-min. Compound **8** at *m*/*z* 498 was identified as 6″-*O*-malonyldaidzin which was released after the digestion and fermentation process; whereas compound **20** at *m*/*z* 267 was identified as 7-hydroxy-4′-methoxyisoflavone and found bonded before the digestion process and released after the fermentation process in all PFAE, PFAC and PFEE fractions.

Regarding tannins, two galloyl-hexosides were found at 22.4 and 22.6 min at *m*/*z* 331 and 334, respectively; the first, compound **9**, was found bonded in all fractions after digestion and released after the fermentation process, while compound **10** was found free and bonded before and after the digestion process in all fractions. Also, stilbene isomers were detected at peaks 15 and 17 and eluted at 25,9 and 26.4 min respectively with *m*/*z* values of 389 which corresponded to resveratrol glucosides which were found bonded to the fiber and released to the supernatant after the digestion and fermentation process.

Flavones were also found bonded and released after the digestion and fermentation process in all fractions. Peak No. 16 which eluted at 26.1 min at *m*/*z* 316 was tentatively identified as 6-methoxyluteolin; peak No. 21 which eluted at 29.0 min at *m*/*z* 312 was identified as 5,4′-dihydroxy-6,7-dimethoxyflavone. Compounds **11** and **12** eluted at 23.2 and 23.5 min and were identified as cyanidin 3-*O*-galactoside at *m*/*z* 448 and epicatechin at *m*/*z* 290. While the flavanol, epicatechin, was detected in all fractions free and bonded, the anthocyanin cyanidin 3-*O*-galactoside was found bonded after digestion and released after the fermentation process in all PFAE, PFAC, and PFEE fractions.

### 3.7. Polyphenolic Quantification

The most abundant phenolic compounds found in extracted, digested and fermented DF fractions were quantified by HPLC-DAD (Limit of quantification, LOQ: 5 µg/mL) (Table 4).

After gastric digestion, a significant decrease (*p* < 0.05) was observed; the results varied from treatments and phenolic compounds. As a result of the alkaline hydrolysis, the most abundant phenolic bounded to the fiber matrix was gallic acid and its highest yield observed before digestion 11,471.5 ± 260 mg/100 g in PFAC and 9131.3 ± 250 mg/100 g in PFEE and after digestion 8042 ± 424 mg/100 g in PFAE; these values were higher than the reported for the Ichida-gaki variety [46].

Phenolic compounds quantified displayed a higher concentration in the undigested fraction, unlike ellagic acid which increased after digestion and the fermentation process in the PFAC fraction, this derived hydroxybenzoic acid was detected in higher concentrations due to the probiotic bacterial fermentation process and the hydrolysis process; previous studies have reported significant increase in yields of ellagic acid because of the solvent effect [47]. *p*-Coumaric acid was also higher in the PFAC fraction, before, after digestion and fermentation. Salicylic acid was found in all samples and fractions and showed a higher concentration in the PFAC.

From the compounds identified by HPLC-DAD-ESI-IT-MS^n^, gallic acid made up to 92% of the total amount of phenolics found in fiber fractions. On the other hand, and in accordance with the recovery and bio-accessibility results, low concentration of bonded phenolics was found to have been released to the supernatant, as a result, gallic acid was quantified after the fermentation process in PFEE and PFAC.

### 3.8. SCFA Profile

SCFAs produced after PFP were quantified in all fractions, before and after the digestion process, and shown in Table 5. The digestion process increased the production of acetic acid; the PFAE fraction presented a higher production of acetic acid before digestion while the PFAC fraction showed a higher concentration of acetic acid when fermenting the fiber matrix after digestion (*p* < 0.05). Propionic acid was produced in lower concentrations before digestion, once digested, it increased its amount significantly (*p* < 0.05); fractions did not show differences in the fermentation production of propionic acid after digestion. Butyric acid was the highest SCFA produced by the probiotic population where the PFAE fraction and the digested fractions showed to be an optimal matrix to produce this compound and allowed the highest yield (*p* < 0.05).

### 3.9. Cytotoxicity Assays

**Figure 3:**
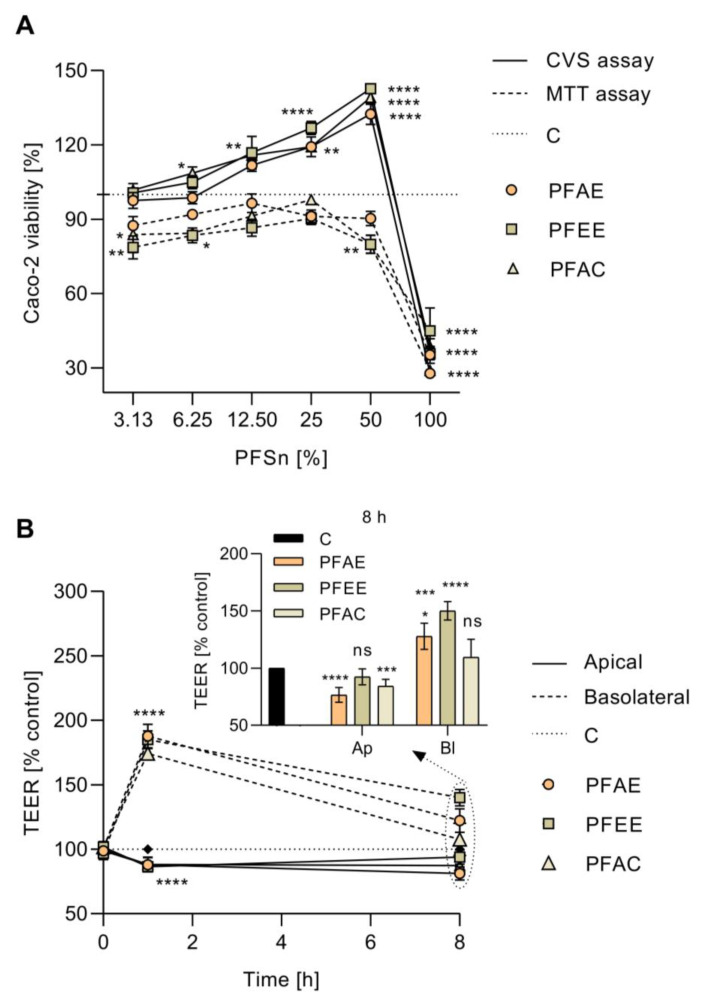
Effect of probiotic fermented supernatants (PFSn) of persimmon fiber (PF) obtained from aqueous extraction (PFAE), ethanolic extraction (PFEE) and acetonic extraction (PFAC) in Caco-2 cells. (**A**) Viability of Caco-2 cells exposed to different concentrations of PFSn of PFAE, PFEE and PFAC by crystal violet staining (CVS) and 3-(4,5-dimethylthiazol-2-yl)-2,5-diphenyltetrazolium bromide (MTT) assays. (**B**) Trans epithelial electronic resistance (TEER) response of Caco-2 cells measured from apical-basolateral (Ap) and basolateral-apical (Bl) directions during 8 h of incubation with PFSn in comparison to untreated monolayers (C) (**** *p* < 0.0001, *** *p* < 0.001, ** *p* < 0.01, * *p* < 0.05, ns *p* > 0.05, ANOVA with Dunnett’s post hoc test).

The viability results of Caco-2 cells in interaction with probiotic fermented supernatants (PFSn) were displayed in Figure 3A. PFAE, PFEE and PFAC fractions were applied; these fractions contained 2.19 ± 0.53, 2.09 ± 0.45 and 1.97 ± 0.41 mg/mL of total SCFAs in the supernatants PFAE, PFEE and PFAC respectively, and were diluted at 50, 25, 12.5, 6.25 and 3.13% of their initial concentration in DMEM.

After incubation, the MTT assay showed that the fermentation supernatant fractions did not exhibit cytotoxic effect in Caco-2 cells exposed from 6.25 to 50% of PFSn. However, a significant reduction of Caco-2 cellular viability (*p* < 0.0001) was recorded in cells exposed to pure fractions. Among them, PFAC supernatant stimulated the lowest Caco-2 cell viability (28.94 ± 6.02%), followed by PFAE (35.30 ± 8.08%) and PFEE (44.88 ± 1.73%), when compared to untreated cells. A 20% reduction of Caco-2 cell viability was also detected at 3.13% of PFAC (*p* < 0.05) and PFEE (*p* < 0.01) when compared to untreated cells.

CVS results (Figure 3A) also confirmed the absence of cytotoxic effects from PFSn in Caco-2 cells exposed from 3.13 to 50% of fermented fractions. Moreover, an increase of Caco-2 cell viability was recorded, especially at 50% of fermented samples (*p* < 0.0001). PFEE stimulated the highest Caco-2 cell proliferation, followed by PFAC and PFAE. Alike the MTT assay, when cells were exposed to pure supernatants, a significant decrease of viability was recorded (*p* < 0.0001).

### 3.10. TEER Response of Cell Culture

Regarding the TEER response of Caco-2 cell monolayers incubated with PFSn, it was found a significant decrease of TEER response of the monolayer when supernatants were added apically after 1 h of incubation (*p* < 0.001) (Figure 3B). After 8 h of incubation, TEER response of PFEE-treated cells was similar to untreated cells (93.89 ± 5.61%, *p* > 0.05) while a reduction of TEER response was recorded in PFAC (87.46 ± 4.71%, *p* < 0.01) and PFAE-treated cells (81.21 ± 5.71%, *p* < 0.001). Contrary, TEER values were significantly higher when PFSn were added in basolateral chamber (*p* < 0.0001), especially after 1 h of incubation. After 8 h of incubation, PFEE-treated cells presented the highest TEER response (140.16 ± 6.48%, *p* < 0.0001), followed by PFAE (122.32 ± 9.16%, *p* < 0.001) and PFAC (113.92 ± 0.93%, *p* < 0.01).

## 4. Discussion

Our results suggest that after appropriate treatment on persimmon fruit byproducts, the bonded metabolites and functional properties were significantly stimulated by the solvent applied; moreover, we found that the obtained DF fractions acted as a nutrient source for beneficial bacteria. Byproducts of this metabolization SCFAs were obtained and interacted with the epithelial cell barrier where they displayed a protective function.

Physicochemical parameters studied in all treatments showed slightly differences influenced by the solvent applied. The most significant TDF value was recorded in PFAC fractions, which were higher than values recorded in persimmon flours [48]. The 65% proportion of IDF in TDF in all extracted fractions of fiber from persimmon byproduct was alike other reports from persimmon byproducts [49], which suggests that the solvent applied did not promote a higher insoluble portion. While some studies have reported a higher SDF/IDF ratio to provide the appropriate physiological effects and bio-accessibility [50], others are reporting increasing evidence of the relationship between IDF and gut health [51]. Due to several logistic challenges, the maturity of processed fruits often fluctuates. Therefore, the physiochemical composition can vary widely; as a result, SDF and TDF content and ratio as well as other nutrients concentration may vary, mostly due to the ripening stage of the processed fruits. Over-ripening has been reported to polymerize tannins in persimmon fruits, and to increase TSS which implies a loss in astringency but also fruit firmness [52,53]. These effects may result in a lower DF yield but with an increased quantity of bonded compounds. These challenges, altogether with environmental conditions may also generate diverse outputs in the extraction and characterization of PF and should be acknowledged when processing byproducts.

After SAE, all fractions seemed to have a low risk for deterioration by microorganisms, enzymatic or physical reactions. Moreover, in adequate conditions, bonded bio-compounds may seem to act as antimicrobial and prebiotic agents themselves. However, byproducts generated by manufacturers tend to be spoiled or partly fermented which will also affect the chemical, microbial composition, and compromise safety of use for human consumption. These issues must be addressed through byproduct quality control and appropriate processing of obtained byproducts prior food upcycling.

Regarding techno-functional properties, WHA and SA are directly implied in health and nutrition as they may promote a satiety effect when swelling and holding water during digestion processes. This behavior in WAA and WHA has been reported for commercially available prebiotics; moreover, obtained results were above intervals reported for persimmon flours (12.19 g/g) and for other fruit byproducts such as lemon (14.4 g/g), orange (9.9 g/g), peach (14 g/g) or apple fiber (15.4 g/g) [45,48]; especially, values obtained from PFAC fractions. Both EA and ES results show the potential application of these fibers to the decrease in the interfacial tension among the hydrophobic and hydrophilic compounds in the food matrix and during the digestion and colonic fermentation process.

OHA was similar to treated persimmon flours obtained from high hydrostatic pressure [54]. Given the strong relation recorded between SDF and TDF with OHA (r = 0.99); DF fractions might derive from a higher availability of hydrophobic bonds within the fiber in all treatments. Moreover, OHA values in all fractions showed a potential function as an emulsifying or stabilizing agent ingredient in fatty matrices as they could help prevent an over greasy incorporation and reduce fat content. On the other hand, GFA, which affects texture and mouthfeel of food matrices, was similar to values for cassava flour (4%) [55]; given GFA’s strong correlation with SDF content in DF fractions (r = 0.99), persimmon extracted fibers could, in addition to provide functional benefits, act as gel forming agents in matrices which require thickening or gelling.

The BHA has been strongly related with the presence of fiber bounded phenolic compounds and linked with the modulation of glucose blood levels, reduction of cholesterol and the conversion of cholesterol to bile acids in the liver to reduce glycemic and lipidemic levels [46,56,57]. In addition, the OHA of fiber fractions which is an essential parameter in the characterization of DF was relatively higher than values reported for persimmon flours [45,48]. Like OHA values, FOB might imply a higher availability of hydrophobic bonds within the PFAC fiber organizational structure. This property shows the capacity of fiber to adsorb or retain oil/fat in its matrix, simulating the conditions of food digestion.

TPC recovery index results showcased statistical differences after digestion process with PFEE fraction with a remarkable decrease. The significant reduction after fermentation of recovery index in PFEE and PFAC fractions are similar to the reports from other food matrices [58]. These results imply a high impact from the extraction treatment in the digestion and fermentation processes and in the bioactive compounds bonded to the fibers, as a result, the PFAE fraction was able to reduce the impact of the digestion process in the reduction, degradation or polymerization of phenolic compounds while allowing the availability of bio-compounds for gut bacteria consumption. On the other hand, results related to bio-accessibility indexes after fermentation are mainly implied with bacterial enzyme action on fiber polysaccharides and the release of bonded phenols [59,60,61].

The fractions of bioactive compounds released from the fiber matrices and found in the supernatant fractions are directly implied in the availability of these compounds during the digestion process and the gut fermentation process for its absorption into the bloodstream. The probiotic bacterial population used in this assay allowed a significant amount of phenolic compounds to be released from the fiber matrix compared to the index of phenolic compounds released after the digestion process.

Assessing TPC by the Folin–Ciocalteu reagent has been also acknowledged as nonspecific to phenolic compounds [37,38]. Even though fiber fractions may present low quantities of interfering compounds, the reagent can be reduced by other compounds present in in vitro digestion, and fermentation such as amino acids, peptides, and reduction sugars. Despite limitations of the TPC assay with the Folin–Ciocalteu reagent, it is an accessible, simple, and reproducible tool when exploring phenolic antioxidants and its reducing capacity. More insightful analyzes through enzymatic reactions, chromatographic and spectrometric quantitation assays should be performed to avoid bias and confirm results.

Regarding to recovery and bio-accessibility indexes of carotenoids, results imply a lower proportion, and concentration of carotenoids released from the fiber matrix, a low stability of both carotenoids and phenolics after the digestion process. Despite a lower recovery index, bio-accessibility index in carotenoids was higher than the phenolic fraction; however, it remained low compared to the TCC bonded to the fibers. Studies have reported persimmon byproducts as a remarkable source of carotenoids, specially β-carotene, lycopene and β-cryptoxanthin. Bonded bio-compounds act as a source of nutrients for gut bacteria, where the fiber matrix acts as a protective agent against the digestion process and allows bonded material to be released by the gut bacteria [60].

The selected bacterial population used in this assay did not allow a significant carotenoid amount to be released from the fiber matrix (<0.6%), in fact, results imply these populations might have consumed some of the carotenoids present in the matrix. It is important to address the high concentration of phenolic compounds and carotenoids bonded to the fiber matrix, while many approaches have been made to integrate food byproducts into food matrixes, taking into consideration the high quantity of these bio-compounds, few have assessed the extractability or availability of them after digestion and fermentation process. Hence, we provide information about the low extractability of these compounds’ despite of previous treatment. As a result, in order to increase the release of these bonded compounds from the fiber matrices, multiple approaches and technologies have to be implemented takin into account the functional properties of DF, the stability of bio-compounds, and the biological interaction of the outcomes.

As regards to the antioxidant activity provided by the bio-compounds presented in fiber matrix, after digestion, the pellet formed showed a lower antioxidant activity than the supernatant fraction, which displays a complex interaction between the soluble fraction and the fiber matrix that formed the pellet. Similar values were reported for the antioxidant activity in soluble fractions and pellets after fermentation. This behavior in the antioxidant activity has been reported in ABTS^•+^ assays in other food matrices [58].

Many studies have reported a similar distribution of antioxidants and bio-compounds in both pellets and soluble fractions, while others have reported contradictory results [48]. Probably, the variability of these results is based on the fiber matrix composition, and the sample preparation methodology, while some authors only use organic solvents for the extraction of bio-compounds, others also modify conditions such as pH, temperature and pressure in order to obtain highest yield of bonded bio-compounds present in the food matrix.

After hydrolysis, the major polyphenolic compound detected and quantified was gallic acid. This hydroxybenzoic acid has been reported to be bonded to the fiber matrix. However, our results suggest that it was released from the galloylated tannins reported and detected in persimmon’s non-extractable fractions [46]. Tannins present in persimmon contain gallic acid residues linked with glucose via glycosidic bonds. The hydroxyl group of both glucose and gallic acid can be considered as the potential interacting sites for the formation of hydrogen bonds with cellulose and hemicellulose. Moreover, tannins can interact with carbohydrates non-covalently or covalently which influences in the extractability of phenolic compounds [62] in persimmon DF. These effects yielded a high concentration of gallic acid after hydrolysis elucidates the complex configuration of persimmon DF.

Soluble tannins have been assessed in variety ‘Rojo Brillante’ due to the formation of salivary protein complexes resulting in astringent sensations [8]. This must be accounted when introducing persimmon DF from byproducts in food matrixes. Additionally, flavonoids coupled with hydrolysable gallic acid moiety through carbon-carbon linkage were also substantial phytochemical moieties bonded in the persimmon DF matrix after SAE. Few differences were assessed in the composition of bonded polyphenols as a result of the solvent applied; whereas the digestion and fermentation processes displayed and released a higher variety of bioactive compounds.

Chemical characteristics of phenolic compounds, such as solubility, hydrophobicity, molecular weight, or configuration are evidently affected by the course of the digestion [63] and fermentation processes. Effects and variability displayed on each phenolic compound are related to its configuration through the fiber matrix and its bond with other carbohydrates and in agreement with the data shown for the bio-accessibility indexes, similarly to other studies reported [64]. Results show the release of these phenolic compounds to the colon where they may undergo metabolism and transformation by bacterial populations into absorbable and beneficial metabolites like SCFAs.

Beneficial microflora plays an important role in metabolization of non-digestible carbohydrates and polyphenols. Both have an important role in the protection of intestinal tract because they keep their antioxidant activity, generate SCFAs and are subsequently set off. From SCFAs, acetic acid, propionic acid, and butyric acid have been among the most documented metabolites because of their health implications [16,65]; PFAE, PFEE and PFAC fractions after digestion were tested as a potential prebiotic matrix with beneficial bacteria that has been reported for the SCFAs metabolization after fermentation. The results varied from each fraction before and after digestion, the SCFA production followed the order propionate > acetate > butyrate, persimmon fibers showed a higher production of SCFAs than the reported for orange, mango, and pear byproducts [66].

Results imply the utilization of the fiber matrix to produce SCFAS is dependent of the digestive process and the application of solvent treatments in the fiber present in the food matrix. We acknowledge the complex composition and outcomes of the gut microbiota fermentation; for these reasons, the main gut beneficial bacteria were tested. However, these results must be confirmed with entire gut microbiome in vitro and in vivo.

PFP is known to exert health benefits in colonic epithelium such as an enhancement of barrier function [67]. Therefore, the interaction of PFSn was determined. Pure fermented fractions of PFAE, PFAC, PFEE decreased the viability of Caco-2 cells according to CVS and MTT assays, suggesting that metabolites produced by PFP contained substances that might either directly inhibit cell-proliferation or inactive it due to alteration of microenvironment, lowering pH or scavenging reactive oxygen intermediates [68], especially as it was the only bio-accessible nutrient in the microenvironment. This effect has also been recorded in PFSn by lactic acid bacteria or Bifidobacterium spp., with cytotoxic effects in HT-29, SW-480 and Caco-2 cell lines [67,69].

Regarding cells incubated with fermented fractions and DMEM, the CVS assay showed no cytotoxic effect of these samples in cell viability; in fact, Caco-2 cell viability increased in the presence of DMEM, dose-dependent. Interestingly, a low decrease of Caco-2 cell viability was observed by MTT assay. Although not significant (*p* > 0.05), fermented samples from 6.25 to 50% of purity may interfere with MTT or succinate dehydrogenase activity, with a loss of viability as an outcome. In addition, it has been reported a direct reduction of MTT to formazan by the interference of phenolics which were detected in PFSn from fiber fractions [70]. The outcome is related to a slightly decrease in Caco-2 viability which may explain the obtained results. Even so, the viability recorded by MTT correlated with those obtained by CVS, indicating that the PFSn from fiber fractions were no cytotoxic and promoted Caco-2 cell viability when compared to untreated cells.

Moreover, it was determined that fermented fractions generated a significant increase of TEER values when added basolateral-apical after 1 and 8 h of incubation (*p* < 0.0001), with the highest TEER value recorded by PCAE and PFEE supernatants. The TEER response of Caco-2 cell monolayer to fermented samples may be due to the presence of SCFAs which have been identified in fermented fractions, especially butyrate in PFAE (1.29 ± 0.02 mg/mL), PFEE (1.10 ± 0.00 mg/mL) and PFAC (1.01 ± 0.00 mg/mL). It has been reported that butyrate increases TEER values of Caco-2 cell monolayers [33] which suggests that PFAE and PFEE (samples with the highest butyrate concentrations) improved the intestinal barrier function of Caco-2 cell monolayers when compared to untreated monolayers, after 8 h of incubation. On the other hand, lower TEER values were recorded by fermented samples when added apical-basolateral, especially by PFAE supernatants after 8 h of incubation. When added apical-basolateral, PFSn interacted directly with intracellular junctional complexes (tight junctions, gap junctions, adherence junctions and desmosome) [71]. The modulation of SCFAs in Caco-2 cell monolayers has been reported to produce lower TEER values [72,73,74], which may increase the permeability of another compounds. Overall, PFSn especially from PFEE and PFAE, showed the potential to improve barrier function in Caco-2 cell monolayers, which has been related to restrictions of the channel from the lumen and into the systemic circulation (abluminal) of larger potentially toxic compounds, as well as allowing the absorption of nutrients, electrolytes and bio-compounds [73].

## 5. Conclusions

This study focused on the solvent extraction effect of DF from persimmon byproducts on its physiological, technological, and prebiotic features. The in vitro gastrointestinal digestion and probiotic bacterial fermentation decreased TPC and TCC and therefore the antioxidant activity of DF. Hydrolysis of covalently bonded compounds in persimmon fiber yielded a high amount of gallic acid. Moreover, metabolites produced by bacterial fermentation were no cytotoxic for human epithelial cells. Overall results show the biological potential of persimmon’s DF is dependent on the SAE process and may promote a strong probiotic response and modulate the epithelial barrier function in a Caco-2 cell model. The underlying mechanism will be further investigated in the future works. These findings contribute to existing knowledge of persimmon byproducts as a DF and bound phenolics source and provide a new insight to its suitability.

## Figures and Tables

**Table 1:** Solvent effects on the physicochemical characteristics of extracted dietary fiber from persimmon byproduct.

Treatment	PFAE	PFEE	PFAC
Protein	0.0006 ± 0.0001 ^a^	0.0014 ± 0.0000 ^a, b^	0.0021 ± 0.0001 ^b^
Ashes	0.08 ± 0.00 ^a^	0.16 ± 0.00 ^b^	0.04 ± 0.00 ^a^
pH	5.50 ± 0.02 ^a^	6.72 ± 0.01 ^b^	6.83 ± 0.02 ^c^
TSS *	1.17 ± 0.29 ^a^	20.33 ± 0.29 ^b^	21.17 ± 0.29 ^b^
IDF	0.62 ± 0.13 ^a^	0.68 ± 0.05 ^a^	0.64 ± 0.08 ^a^
SDF	0.19 ± 0.02 ^a^	0.14 ± 0.03 ^a^	0.30 ± 0.16 ^b^
TDF	0.82 ± 0.11 ^a^	0.81 ± 0.08 ^a^	0.94 ± 0.08 ^b^

Values expressed as g/g of sample. Significant differences in physicochemical characteristics were determined in persimmon fiber (PF) obtained by aqueous extraction (PFAE), ethanolic extraction (PFEE) and acetonic extraction (PFAC) (*p* < 0.05, ANOVA. Different letters near values in the same row indicate significative differences according to Tukey’s post hoc test). TSS *, total soluble solids (°Brix); IDF insoluble dietary fiber; SDF soluble dietary fiber, TDF, total dietary fiber.

**Table 2:** Effect of in vitro gastrointestinal digestion and probiotic fermentation processes in the antioxidant activity of persimmon fiber by colorimetric ABTS^•+^ and DPPH radical scavenging assays.

		ABTS^•+^	DPPH
Samples	Treatments	mg Trolox/g Sample	mg Trolox/g Sample
Extracted fiber	PFAE	2.81 ± 0.34 ^a^	2.12 ± 0.13 ^a^
	PFEE	2.71 ± 0.09 ^b^	2.04 ± 0.09 ^b^
	PFAC	2.71 ± 0.05 ^b^	2.04 ± 0.04 ^b^
Digested fiber	PFAE	1.21 ± 0.11 ^f^	0.91 ± 0.11 ^f^
	PFEE	1.43 ± 0.30 ^e^	1.08 ± 0.09 ^e^
	PFAC	1.05 ± 0.09 ^g^	0.79 ± 0.19 ^g^
Supernatant digested fiber	PFAE	1.68 ± 0.11 ^d^	1.26 ± 0.11 ^d^
	PFEE	1.68 ± 0.09 ^c, d^	1.26 ± 0.09 ^c, d^
	PFAC	1.68 ± 0.05 ^c, d^	1.26 ± 0.05 ^c, d^
Fermented fiber	PFAE	0.73 ± 0.12 ^h^	0.55 ± 0.25 ^h^
	PFEE	0.67 ± 0.32 ^i^	0.51 ± 0.12 ^i^
	PFAC	0.61 ± 0.13 ^j^	0.46 ± 0.15 ^j^
Supernatant fermented fiber	PFAE	1.68 ± 0.10 ^c, d^	1.26 ± 0.10 ^c, d^
	PFEE	1.68 ± 0.13 ^c, d^	1.26 ± 0.13 ^c, d^
	PFAC	1.68 ± 0.22 ^c^	1.26 ± 0.22 ^c^

The antioxidant activity of extracted persimmon fiber by aqueous extraction (PFAE), ethanolic extraction (PFEE) and acetonic extraction (PFAC) decreased after digestion and fermentation of persimmon fiber (*p* < 0.05, ANOVA. Different letters in the same column indicate significant differences among samples according to Tukey’s post hoc test).

**Table 3:** Identified compounds in treated persimmon fiber fractions by HPLC-DAD-ESI-IT- MS^n^.

No.	RT (Min)	HPLC-DAD UV−vis Spectrum λmax (nm)	[M−H]− *m*/*z*	HPLC-DAD-ESI-IT-MS^n^ Experiments *m*/*z*	Compound Identity	Molecular Formula	Extracts Present
**1**	7.7	272/280	126.1 (100) 170.5 (57)	MS^2^ [170.5] 126.1 (100) 168.4 (31) 124.1 (15.4)	Gallic acid *	C_7_H_6_O_5_	U1, U2, U3, DP1, DP2, DP3, FP1, FP2, FP3
**2**	16.0	305/272	164.5 (100)	MS^2^ [164.5] 120.1 (100)	*p*-Coumaric acid *	C_9_H_8_O_3_	U1, U2, U3, DP1, DP2, DP3, DS3,
**3**	16.1	260/272	303.7 (100)	MS^2^ [303.7] 301.6 (100) 259.2 (60) 186.5 (16.9)	Ellagic acid *	C_14_H_6_O_8_	U1, U2, U3, DP1 DP2, DP3, DS1, DS2, DS3
**4**	16.6	320/272	209.8 (100) 225.0 (45)	MS^2^ [209.8] 165.4 (100) 150.3 (60) 164.4 (22)	3,5-Dimethoxy-4-hydroxycinnamic acid *	C_11_H_12_O_5_	U1, U2, U3, DP1, DP2, DP3, DS1, DS2, DS3
**5**	18.7	305/280	301.6 (100) 241.1 (94) 138.2 (76)	MS^2^ [301.6] 138.2 (100) 139.2 (10) 94.0 (4)	Salicylic acid *	C_7_H_6_O_3_	U1, U2, U3, DP1, DP2, DP3, DS1, DS2, DS3
**6**	20.8	270/332	514.3 (100)	MS2 [514.3] 170.9 (100) 342.1 (31)	3,5-Dicaffeoylquinic acid **	C_25_H_24_O_12_	DP1, DP2, DP3
**7**	20.9	270/332	1021.3 (100) 948.1 (42) 194.7 (42)	MS^2^ [948.6] 888.5 (100) 909.7 (47) 930.1 (42)	Spinacetin 3-*O*- (2″-*p*-coumaroylglucosyl) (1->6)-[apiosyl (1->2)]-glucoside **	C_43_H_48_O_24_	U1, U2, U3, DP1, DP2, DP3, DS1, DS2, DS3, FP1, FP2, FP3
**8**	21.6	270/332	498.3 (100)	MS^2^ [498.6] 350.6 (100) 412.9 (94)	6″-*O*-Malonyldaidzin **	C_24_H_22_O_12_	DP1, DP2, DP3, DS1, DS2, DS3, FP1, FP2, FP3
**9**	22.4	270/332	331.2 (100)	MS^2^ [331.3] 228.9 (100) 293 (96) 210.8 (90)	Galloyl-hexoside I **	C_13_H_16_O_10_	DP1, DP2, DP3, FP1, FP2, FP3
**10**	22.6	270/332	334.4 (100)	MS^2^ [334.4] 316.1 (100) 172.6 (22) 332.2 (16) 287.7 (2)	Galloyl-hexoside II **	C_13_H_16_O_10_	U1, U2, U3, DP1, DP2, DP3, DS1, DS2, DS3, FP1, FP2, FP3
**11**	23.2	270/332	448.3 (100)	MS^2^ [448.3] 384.1 (100) 402.2 (54)	Cyanidin 3-*O*-galactoside **	C_21_H_21_O_11_	DP1, DP2, DP3, DS1, DS2, FP1, FP2, FP3
**12**	23.5	270/332	289.8 (100)	MS^2^ [289.8] 271.6 (100) 142.2 (22) 130.2 (7)	Epicatechin *	C_15_H_14_O_6_	U1, U2, U3, DP1, DP2, DP3, DS1, DS2, DS3, FP1, FP2, FP3
**13**	24.2	280/320	446.3 (100)	MS^2^ [446.1] 249.2 (100)	Kaempferol-7-glucoside **	C_21_H_19_O_11_	DP1, DP2, DP3, DS1, DS2, DS3, FP1
**14**	25.2	280/320	783.5 (100) 391.3 (27.7)	MS^2^ [391.3] 343.1 (100) 170.9 (56)	Quercetin glucoside I **	C_33_H_40_O_21_	DP1, DP2, DP3, FP1, FP2, FP3
**15**	25.9	272/280	389.5 (100)	MS^2^ [389.6] 339 (100) 342.2 (90) 297.2 (43)	Resveratrol glucoside I **	C_20_H_22_O_8_	DP1, DP2, FP1, FP1, FP2, FP3
**16**	26.1	320/360	316.2 (100)	MS^2^ [316.5] 172.6 (100) 297.9 (75) 128.3 (7)	Methoxyluteolin **	C_16_H_12_O_7_	U1, U2, U3, DP1, DP2, DP3, DS1, DS2, DS3, FP1, FP2, FP3
**17**	26.4	272/280	389.2 (100)	MS^2^ [389.2] 369.1 (100) 352.9 (56) 296.1 (38) 343.1 (21)	Resveratrol glucoside II **	C_20_H_22_O_8_	DP1, DP2, DP3, DS1, DS2, U3, FP1
**18**	27.2	280/320	783.6 (100) 391.3 (30)	MS^2^ [391.3] 218.9 (100) 357.1 (92)	Quercetin glucoside II **	C_33_H_40_O_21_	DP1, DP2, DP3
**19**	27.6	280/320	754.2 (100) 718 (95)	MS^2^ [754.2] 718.4 (100) MS^3^ [718.4] 661.4 (100)	Kaempferol 3-*O*-glucosyl-rhamnosyl-galactoside **	C_21_H_20_O_11_	U1, U2, U3, DP1, DP2, DP3, DS1, DS2, DS3, FP1, FP2, FP3
**20**	28.5	280/320	267.6 (100)	MS^2^ [267.6] 98.0 (100) 297.9 (75) 128.3 (7)	7-Hydroxy-4′-methoxyisoflavone **	C_16_H_12_O_4_	U1, U2, U3, FP1, FP2
**21**	29.0	280/320	312.1 (100)	MS^2^ [312.1] 98.0 (100) 310.0 (18) 124.1 (17)	5,4′-Dihydroxy-6,7-dimethoxyflavone **	C_17_H_14_O_6_	U1, U2, U3, DP1, DS1, DS2, DS3, FP1, FP2, FP3
**22**	29.5	320/360	355.2 (100)	MS^2^ [355.2] 265 (100) 291 (56) 234.8 (29)	Ferulic acid glucoside **	C_16_H_20_O_9_	DP1, DP2, DP3
**23**	30.2	320/360	297.2 (100)	MS^2^ [297.4] 277.1 (100) 234.5 (30)	*p*-Coumaroyl tartaric acid **	C_13_H_12_O_8_	DP1, DP2, FP1

U: Undigested, D: Digested, F: Fermented, P: Pellet, S: Supernatant, 1: Persimmon Fiber Aqueous Extraction (PFAE), 2: Persimmon Fiber Ethanolic Extraction (PFEE), 3: Persimmon Fiber Acetonic Extraction (PFAC). ^*^ Authentic standards. ^**^ Tentatively identified.

**Table 4:** Quantification of the most abundant polyphenolic compounds found in treated persimmon fiber fractions.

		Phenolic Compounds (mg/g or mL Sample)				
Samples	Treatment	Gallic Acid	Sinapic Acid	*p*-Coumaric Acid	Salicylic Acid	Ellagic Acid
Extracted fiber	PFAE	58.63 ± 0.46 ^c^	0.69 ± 0.04 ^b^	0.57 ± 0.01 ^d^	2.19 ± 0.18 ^b^	1.26 ± 0.01 ^c^
	PFEE	91.31 ± 2.45 ^b^	0.69 ± 0.04 ^b^	0.14 ± 0.01 ^d^	2.18 ± 0.15 ^b^	1.89 ± 0.19 ^b^
	PFAC	114.72 ± 2.60 ^a^	0.77 ± 0.08 ^a^	3.73 ± 0.22 ^b^	2.24 ± 0.09 ^b^	2.05 ± 0.08 ^b^
Digested fiber	PFAE	80.42 ± 4.24 ^b^	0.46 ± 0.00 ^d^	1.28 ± 0.08 ^c^	3.57 ± 0.18 ^a^	1.03 ± 0.05 ^d^
	PFEE	64.94 ± 3.32 ^c^	0.34 ± 0.03 ^f^	0.08 ± 0.00 ^e^	1.16 ± 0.09 ^e^	1.90 ± 0.11 ^b, c^
	PFAC	104.20 ± 9.38 ^a^	0.67 ± 0.03 ^b^	3.57 ± 0.18 ^b^	1.35 ± 0.11 ^e^	4.22 ± 0.13 ^a^
Supernatant digested fiber	PFAE	<0.001	<0.001	<0.001	<0.001	<0.001
	PFEE	<0.001	<0.001	<0.001	<0.001	<0.001
	PFAC	<0.001	<0.001	<0.001	<0.001	<0.001
Fermented fiber	PFAE	33.06 ± 2.64 ^d^	0.51 ± 0.01 ^d^	<0.001	1.87 ± 0.15 ^c^	1.40 ± 0.06 ^c^
	PFEE	50.04 ± 0.97 ^c^	0.47 ± 0.04 ^d^	<0.001	1.71 ± 0.14 ^d^	2.01 ± 0.04 ^b^
	PFAC	57.01 ± 3.04 ^c^	0.57 ± 0.01 ^c^	4.56 ± 0.09 ^a^	2.20 ± 0.11 ^b^	4.59 ± 0.37 ^a^
Supernatant fermented fiber	PFAE	<0.001	<0.001	<0.001	<0.001	<0.001
	PFEE	0.006 ± 0.00 ^e^	<0.001	<0.001	<0.001	<0.001
	PFAC	0.004 ± 0.00 ^e^	<0.001	<0.001	<0.001	<0.001

Phenolic compounds present in persimmon fiber by aqueous extraction (PFAE), ethanolic extraction (PFEE) and acetonic extraction (PFAC) decreased significantly in the fermented samples. Gallic acid was the most abundant phenolic in samples, especially PFAC (*p* < 0.05, ANOVA. Different letters in the same column indicate significant differences among samples according to Tukey’s post hoc test).

**Table 5:** Short chain fatty acid (SCFA) profile of probiotic fermented supernatants from treated persimmon fiber fractions.

SCFA (mg/L)	Treatment	Undigested Fraction	Digested Fraction
Acetic acid	PFAE	0.65 ± 0.01 ^a, b^	0.67 ± 0.01 ^a, b^
	PFEE	0.65 ± 0.00 ^a^	0.78 ± 0.01 ^c^
	PFAC	0.68 ± 0.01 ^b^	0.76 ± 0.01 ^c^
Propionic acid	PFAE	0.09 ± 0,00 ^a^	0.23 ± 0.01 ^b^
	PFEE	n.d.	0.21 ± 0.01 ^b^
	PFAC	0.20 ± 0.02 ^b^	0.20 ± 0.01 ^b^
Butyric acid	PFAE	0.95 ± 0.01 ^a^	1.29 ± 0.02 ^d^
	PFEE	1.03 ± 0.00 ^b^	1.10 ± 0.00 ^d^
	PFAC	1.06 ± 0.00 ^c^	1.01 ± 0.00 ^b^

Significant differences were determined among persimmon fiber samples treated by aqueous extraction (PFAE), ethanolic extraction (PFEE) and acetonic extraction (PFAC). In vitro gastrointestinal digestion process stimulated significant differences in propionic and butyric acids (*p* < 0.05, ANOVA. Different letters near values indicate significant differences among all samples according to Tukey’s post hoc test).

## Data Availability

Data is contained within the article.
